# High-Dose Neutrophil-Depleted Platelet-Rich Plasma Therapy for Knee Osteoarthritis: A Retrospective Study

**DOI:** 10.3390/jcm13164816

**Published:** 2024-08-15

**Authors:** Andrea De Matthaeis, Maria Bianchi, Rossana Putzulu, Giulio Maccauro

**Affiliations:** 1 Department of Orthopedics and Traumatology, Fondazione Policlinico Universitario Agostino Gemelli IRCCS, Università Cattolica del Sacro Cuore, 00168 Rome, Italy; giulio.maccauro@policlinicogemelli.it; 2 Dipartimento di Diagnostica per Immagini, Radioterapia Oncologica ed Ematologia, Fondazione Policlinico Universitario “A. Gemelli” IRCCS, 00168 Rome, Italyrossana.putzulu@policlinicogemelli.it (R.P.)

**Keywords:** platelet-rich plasma, knee osteoarthritis, cartilage regeneration, regenerative medicine

## Abstract

**Background/Objectives**: Encouraging results have been reported for Platelet-Rich Plasma (PRP) treatment for knee osteoarthritis (KOA). This study reports the efficacy and safety of a high dose of neutrophile and red-blood-cell-depleted PRP to treat patients with KOA. **Methods**: A total of 212 consecutive patients diagnosed with Kellgren–Lawrence (KL) grading 1–3 KOA chronic knee pain for at least 1 year were treated with three injections at 15-day intervals with a high dose of neutrophil-depleted PRP (4 billion platelets). Clinical outcomes were retrospectively recorded as the percentage of responders at 3-, 6-, and 12-month follow-up, following the OMERACT-OARSI criteria. Pain, through the VAS score and WOMAC score, was also been recorded. **Results**: A total of 4 mL of PRP containing 4 × 10^9^ platelets was obtained by single-spin centrifugation and injected intra-articularly into each patient with no preactivation. The overall responder rate of patients responding to the OMERACT-OARSI criteria at 3, 6, and 12 months was 68.9%, 72.7%, and 70.6%, respectively. A significant improvement in VAS and WOMAC scores at 3-, 6-, and 12-month follow-up compared to the pretreatment value (*p* < 0.01) was observed. The lowest VAS score was observed at 6 months overall and in all three KL-graded groups. The KL2 groups showed the best results regarding pain reduction and their WOMAC score at 6 months (*p* < 0.01). **Conclusions**: For KL1–3 KOA, a high dosage of neutrophil-depleted PRP is a successful treatment. It has long-lasting effects that last up to one year, relieves symptoms, and may slow the advancement of the disease.

## 1. Introduction

Osteoarthritis (OA) is a cartilage degenerative pathology associated with both structural and functional changes in the joint, affecting more than 500 million people worldwide, with an impressive increase in cases due to the aging of the population [1]. Osteoarthritis does not simply result from regular wear and tear but instead arises from a multifaceted interaction of genetic, biomechanical, and inflammatory elements [2]. The gradual breakdown of cartilage, changes in subchondral bone density, and the initiation of synovial inflammation all have roles in the development of knee osteoarthritis. Growth factors, adipokines such as leptin, chemokines, proinflammatory and anti-inflammatory cytokines, and growth factors interact to mediate these complex alterations [2]. Another interesting aspect of the disease’s progression is the epigenetic variations able to regulate genetic expression through DNA methylation, histone modifications, and mRNA [2]. 

For over 30 years, Platelet-Rich Plasma (PRP) has been utilized for diverse purposes, indicating a potential autologous therapy in the field of regenerative medicine [3,4]. Recently, the European Society of Sports Traumatology, Knee Surgery and Arthroscopy (ESSKA) consensus indicated PRP as a valid treatment option for knee OA (KOA), based on a growing body of evidence in the existing literature [5]. PRP therapy is the most frequently used autologous orthobiologic therapy for KOA because it is minimally invasive, repeatable, and safe [5]. Over the past ten years, a great deal of research has been conducted to create non-surgical therapy alternatives that might potentially replace or delay more involved treatments like total knee arthroplasty (TKA) [6]. Recently, PRP treatment has been evaluated as an effective option for TKA for direct patient benefit and to alleviate health systems in particular situations, such as the pandemic [7,8]. A retrospective query was run on a database of 160 million records in the United States from 2016 to 2022, comparing the percentage of patients undergoing a later TKA after PRP, hyaluronic acid (HA), and corticosteroid (CS) injections [8]. In the PRP cohort, only 2.2% (71 patients) out of 3240 patients underwent a later TK, while in the CS and HA cohort, 5.9% and 8% underwent a later TKA, respectively (CS cohort: 81,271 out of 1,382,572 patients; HA cohort: 13,044 out of 164,000 patients) [8]. The average time to TKA from first injection in the HA group was 377.8 days and in the CS group it was 370.0 days; with CS and HA at 4 years post-injection, the same proportions of TKA-free survival occurred (*p* = 0.05) [8]. Unfortunately, the PRP group was removed from the comparison on time to TKA because of its low population size. Positive outcomes following PRP treatment are thought to be mostly attributed to the anti-inflammatory properties of PRP due to chronic inflammation’s correlation with osteoarthritis [9,10,11]. PRP has a dual effect on articular cartilage injuries. Firstly, it inhibits Nuclear Factor kappa B (NFκB) signaling, thus reducing inflammation. Second, it increases chondrocyte secretion of anabolic factors such as Transforming Growth Factor beta (TGF-β1) and Interleukins IL-4, IL-10, and IL-13, which enhance extracellular matrix synthesis and cartilage regeneration [4,12,13]. It is well known that growth factors secreted by platelet alfa-granules have an essential role in tissue regeneration. In 1987, Sprugel et al. [12] provided in vivo evidence that PDGF, bFGF, and TGF beta induce granulation tissue. The responses that were observed after 10 days were found to represent a secondary process that is primarily mediated through the recruitment of effector immune cells, macrophages, and lymphocytes, which are drawn into the tissue by each growth factor [12]. Therefore, the effects observed were not directly caused by the factors themselves. Moreover, platelets release chemokines, cytokines such as platelet factor 4 (PF4), P-Selectins (integrin activators), and RANTES, which are responsible for recruiting and activating other immune cells [4]. Additionally, PRP releases Tissue Inhibitors of Metalloproteases (TIMPs), which inhibit the increased expression of MMP-1 and MMP-3 induced by inflammatory factors, thereby decreasing extracellular matrix degradation and improving the condition of articular cartilage injury [14]. These data are in accordance with clinical outcomes, which indicates that Leukocyte-Poor PRP (LP-PRP) may be superior to Leukocyte-Rich PRP (LP-PRP) to treat KOA [15,16,17]. Furthermore, it is widely recognized that PRP composition ought to have minimal or absent red blood cells (RBCs) to prevent the harmful impact of hemolysis and eryptosis on musculoskeletal tissues [18]. Furthermore, the analgesic impact of LP-PRP appears to be larger than that of LR-PRP, as indicated in a recent meta-analysis of 24 RCTs involving 1344 patients with knee OA [19]. Moreover, the data indicate that multiple injections can outperform a single injection at 1 year follow-up [5]. More importantly, the effectiveness of PRP seems to be strongly related to a platelet dose higher than 3.5 × 10^9^ platelets, while a lower platelet dose around 1.5 × 10^9^ platelets showed negative results [13]. 

In keeping, KOA patients were treated with a high-dose of Leukocyte-Poor PRP (LP-PRP), containing mainly mononuclear cells at a physiological concentration as the residual WBC population, with no red contamination, able to reach a clinical dose of 4 × 10^9^ platelets in a single-spin procedure. 

Consequently, the current retrospective study might confirm the safety and efficacy of this high-platelet-dose, neutrophil-depleted, autologous PRP for knee OA.

## 2. Materials and Methods

### 2.1. Study Design and Patients

A total of 212 consecutive patients, for a total of 636 infiltrative PRP procedures, were treated for chronic symptomatic knee osteoarthritis in our center between 2021 and 2023. 

The clinical protocol after PRP quality validation was approved by the Transfusional Center, according to the current regulation of non-transfusional blood component (DM 1 August 2019) with the protocol code 859. For this study, the electronic clinical records of this cohort’s patients were reviewed retrospectively. The study was approved by the ethics committee of our hospital. 

All treated patients had knee osteoarthritis based on Kellgren–Lawrence (KL) grades 1–3 and had chronic knee pain for at least a year, despite various known conservative therapies (oral nonsteroidal anti-inflammatory medications, HA, or corticosteroid injections). A standing anteroposterior and lateral radiograph was acquired to assess the grade of KOA using the Kellgren–Lawrence (KL) classification system.

Each patient was assessed with X-ray and a complete blood count before PRP treatment was performed.

The inclusion criteria for patients eligible for the protocol included the following:

- Age: >18 years;
- Hgb: >12 g/dL;
- PLT (minimum value): ≥120,000/µL;
- WBC < 10,000/mm^3^;
- No corticosteroid therapies for more than one month;
- No NSAIDs for at least one week.

The exclusion criteria included sepsis, bacteremia, fever, infectious diseases, immunodeficiency syndromes, hematological diseases, patients on anticoagulant or antiplatelet therapy, severe heart diseases, hemodynamic instability, pregnancy, alcohol abuse, and drug use. Patients diagnosed with tricompartmental OA KL grade 4, rheumatoid arthritis, or concomitant severe hip OA, previously treated with a high tibial osteotomy or cartilage transplantation procedure, were not included. We also excluded patients with infections, necrosis, osteomyelitis, and inflammation at the inoculation site. Patients with a diaphyseal varus deformity of 4° and valgus of 16° that required osteotomy were excluded “ab initio” after evaluation by X-ray of the lower limbs in full leg under load in two antero-posterior and latero-lateral projections. Written informed consent was obtained from all patients. 

#### Data Collection

The clinical outcome was evaluated by recording the visual analog scale (VAS) score (0 = no pain to 10 = worst possible pain) [20]. The functional levels of the patients were assessed by WOMAC, which is considered a reliable and valid method for the assessment of patients with knee OA [21], prior to the first injection and at the 3-, 6,- and 12-month follow-up. 

**Figure 1:**
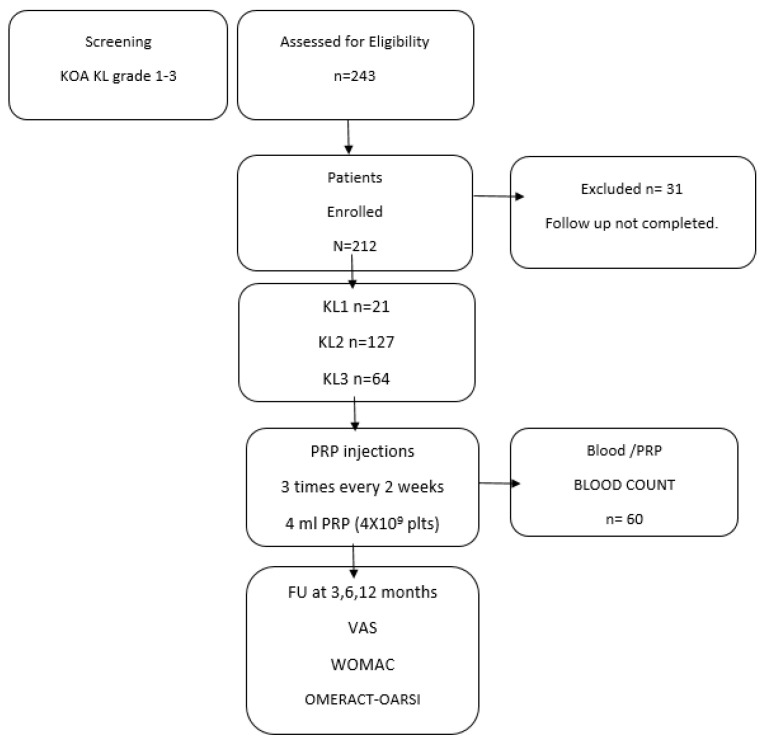
Flowchart of retrospective cohort data collection.

The clinical outcome follow-up of 31 patients was not completed; therefore, we collected complete 12-month follow-up data on 212 patients (Figure 1).

To compare efficacy to prior research on knee OA patients treated with PRP from the same device, we also recorded the OMERACT-OARSI score, based on a combination of absolute and relative changes in pain, function, and patient overall assessment. As in the earlier study by Saita et al. [21], patients were identified as responders if one of the following two criteria was satisfied: (1) significant pain improvement: ≥50% improvement + absolute change of ≥20 or (2) improvement in at least two of the following: ≥20% improvement + absolute change of ≥10 in pain, ≥20% improvement + absolute change of ≥10 in function, or ≥20% improvement + absolute change of ≥10 in the patient’s global assessment of disease activity.

The clinical outcome was assessed at the initiation of treatment and at 3, 6, and 12 months after the third PRP injection. 

### 2.2. PRP Preparation and Protocol

The PRP production protocol and the quality of the produced PRP were certified by the Transfusional Center (protocol code 859). The PRP preparation was prepared using a single-spin centrifugation of whole blood utilizing two Tropocells PRP 11mL Vacutainer tubes (Estar Medical, Israel). These PRP tubes contain a separator gel and a buffered pH 7.2 dextrose solution anticoagulant. A total of 22 mL of whole blood was drawn, and 2 mL of PRP was obtained from each tube after PPP was discarded, according to the manufacturer’s instructions. In brief, 22 mL of whole blood were taken into two Tropocells PRP tubes. Next, the tubes were centrifuged for 10 min at 1500× *g*.

After discarding the supernatant Platelet-Poor Plasma (PPP), 20× gentle mixing was performed to optimize platelet harvesting from the surface of the separation gel; 2.0 mL of PRP from each tube was recovered and pooled together to obtain a final dose of 4 mL of PRP. PRP was immediately injected. PRP was not preactivated with CaCl_2_ or Ca/Gluconate, with the aim of having in vivo activation by the patient’s collagen, in the attempt to release growth factors in the intra-articular space and not before. A blood count and a PRP platelet count were performed to establish the platelet dose.

PRP injection was administered to a supine patient with fully extended knees using an aseptic technique, through the lateral suprapatellar approach into the suprapatellar bursa with a 21-gauge needle inserted 1 cm above and lateral to the superior lateral aspect of the patella, angled at 45 degrees beneath the patella. If joint fluid could be aspirated, it was removed before the injection of PRP. In our protocol, LP-PRP was administered three times, once every 15 days. After PRP treatment, patients were allowed to bear weight, and local ice application was prescribed for 20 min, 4–6 times a day, for the first 48–72 h to reduce discomfort. Activities of the knee were not recommended for 48–72 h.

### 2.3. Statistical Analyses

To test the efficacy of PRP therapy, a Fisher’s exact test was performed to compare the results at the baseline and follow-up end points. Fisher’s exact test was used to compare each KL grade and confirm if the success of PRP therapy was dependent on the severity of KOA. An independent statistician conducted the statistical analysis using SPSS software (SPSS 17.0, SPSS, Chicago, IL, USA). 

The data between the baseline and follow-up times (3, 6, and 12 months) were compared within groups using Student’s t-test and paired or Wilcoxon signed-rank tests, as appropriate. The data from the various follow-up points were evaluated using repeated-measure analysis of variance, followed by post hoc tests. All *p*-values were two-sided, with *p*-values < 0.05 indicating statistical significance.

## 3. Results

### 3.1. Patient Characteristics

Table 1 shows the patients’ demographics.

### 3.2. Biological Characteristics of Injected PRP

Complete blood counts were performed before and after PRP injections on 60 consecutive patients (20 patients for each group: KL1, KL2, and KL3). The baseline platelet count ranged from 179 to 305  × 10^3^ platelet/µL (mean: 225 ± 37 × 10^3^/µL), corresponding to a total of 4.7 × 10^9^ platelets in the drawn blood (4.7 ± 0.79 × 10^9^).

From 20 mL of blood, we obtained 4 mL of PRP (2 mL of PRP from each tube) containing an average platelet concentration of 960 +/− 108 × 10^3^/µL platelets, corresponding to an average platelet dose of 4 × 10^9^ (range: 3.82–4.24 × 10^9^; mean value: 3.9 +/− 0.2). The platelet concentration did not differ across the three KL-grade patients’ groups (PRP KL1 937 +/− 16, KL2 896 +/− 33, and KL3 989 +/− 25). The obtained platelet recovery rate was 82%, and the platelet concentration fold was 4X. Regarding leukocyte content, the PRP obtained was a LP-PRP, with a depletion of neutrophils and a residual white blood cell (WBC) concentration of 3 +/− 0.22 × 10^3^/µL, mostly represented by mononuclear cells (85%). The RBC concentration was less than 0.05 × 10^6^/µL, showing that the separator gel effectively removed RBCs and reversed the initial composition of blood (95% RBCs). The gel separation was stable, and no red cell contamination was detected during mixing for inversion to resuspend platelets in PRP. 

According to the DEPA classification, the BBB a high-dose platelet (B: high dose of injected platelets, from 3 to 5 billion platelets; B: efficiency of platelet recovery rate 70–90%; B: pure PRP percentage of platelets in the PRP compared with the RBC and leucocytes occur from 70% to 90%) [22]. According to the PAW classification system, the obtained PRP is classified as P3-Bβ PRP (P3; platelet concentration from 750,000 to 1,250,000 platelets/mL, no exogen activator, B Leukocyte concentration below baseline level, and β Neutrophil concentration below baseline level) [23]. 

The biological profile of the obtained PRP is reported in Table 2. 

### 3.3. Clinical Outcome after PRP According to OMERCAT-OARSI, VAS, and WOMAC Scores

**Figure 2:**
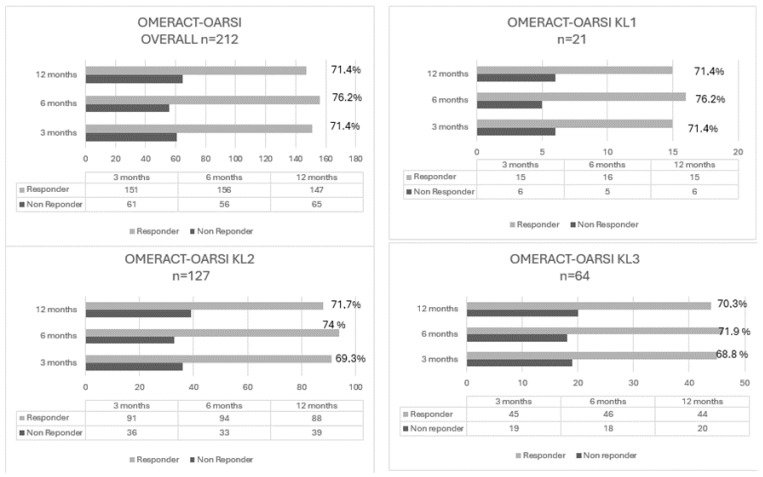
Responder rate (OMERACT-OARSI).

The clinical outcome was recorded 3, 6, and 12 months after PRP treatment. The overall responder rate of patients responsive to the OMERACT-OARSI criteria at 3, 6, and 12 months was 71.2%, 73.6%, and 69.3%, respectively. The highest responder rates were recorded for KL grade 2 at 6 months (76.3%) and 12 months (71.4%). The results are reported in Table 3 and Figure 2.

Table 4 shows a significant improvement in VAS scores at the 3-, 6-, and 12-month follow-up compared to pretreatment values (*p* < 0.001). The lowest VAS score was observed at 6 months, both overall and in all three KL- graded groups. 

At 12 months, the VAS score was higher than at 6 months, even if it was still significatively lower than at the basal level, suggesting that the analgesic effect of PRP started to vanish. The KL2 groups showed the best result regarding pain reduction at the 6-month FU.

A similar trend was observed for the WOMAC total score: there was a significant improvement at the 6- and 12-month follow-up, compared to the pretreatment value (*p* < 0.001).

The best clinical WOMAC total score was recorded at 6 months overall and in all three KL groups. The best clinically significant outcome was observed in all groups at 6 months. 

All three KL groups showed patients with mild transient adverse events like pain and stiffness. No adverse effects were observed in any patients.

## 4. Discussion

PRP has received significant attention lately as a potential Disease-Modifying OA Drug (DMOAD) therapy for symptomatic knee osteoarthritis [5,24,25]. 

The PRP used in this study is classified as P3-Bβ PRP according to the PAW classification system [23], while according to the DEPA classification it is BBB, a high-dose platelet [22].

Our retrospective study on 212 patients demonstrates that a dose of 4 billion platelets (3.9 +/− 0.2) in 4 mL volume of Neutrophil-Poor PRP improves functional outcomes and reduces pain in patients with grade 1–3 KL KOA. Moreover, in our cohort of patients, we observed an overall responder rate, based on OMERACT-OARSI responder criteria, of 71.2%, 73.6%, and 69.3%, respectively, at 3, 6, and 12 months. The most beneficial effects occurred in terms of the number of responder patients, and the clinical outcomes reached their peak after 6 months of the injections and were reduced later at 12 months, even though the outcome remained better compared to the pretreatment baseline level. Our data are in line with those observed in a prospective clinical study on 517 patients with KL2 and KL3 KOA treated with LP-PRP produced by the same PRP device, where 4 to 5 mL of LP-PRP was injected three times every 4 weeks, resulting in a 75.2% responder rate in KL2 and 66.5% in KL3 after 12 months [26]. Saita et al. [26] also treated KL4-grade KOA, which showed a significantly lower responder rate (50.9%) than KL2 and KL3 patients, suggesting that KL4 KOA is a significative predictor of a negative clinical outcome. Based on these data, we decided to exclude KL4 patients and treat KL1 KOA patients instead, which showed a high rate of responders at 6 and 12 months, 76.4% and 71.2%, respectively. 

Our results, in terms of clinical outcome measured as VAS and WOMAC scores, align with previous studies [5,27,28,29]. However, the comparison is challenging due to considerable differences in PRP platelet concentrations, doses, leukocytes, and red blood cell contents. Despite these challenges and confounding factors, a recent meta-analysis of 35 RCTs indicated a better overall outcome in patients treated with PRP compared to control, HA, or steroids at 3-, 6-, and 12-month follow-up intervals, as confirmed in our study [27]. More interesting is comparing our real-world data to RCTs trial data on KOA treated with a comparable PRP in terms of leukodepletion and platelet dose. Patel et al.’s [25] RCT trial compared the efficacy of two different single-injection LP-PRP platelet doses in patients diagnosed with KL1 and KL2 KOA: 4 mL of PRP standard dose containing 2.8 billion/platelet (2.82 × 10^9^) billion and 8 mL of PRP containing 5.5 billion platelets (5.65 × 10^9^). The Leucocyte-Poor PRP used was 3.5 times concentrated, with an average platelet concentration of 706.74 × 10^3^/µL in the standard dose group and 681.44 × 10^3^/µL in the high dose group. Patients with early knee OA experienced significantly superior improvement in pain and function when treated with an 8 mL injection of PRP containing a high platelet dose of 5.5 billion versus a 4 mL injection of PRP containing a lower dose of 2.8 billion platelets [25]. 

Recently, these data were confirmed in a systematic review by Berrigan et al. [30]. Their analysis showed that twenty-eight studies, with a mean platelet dose of 5.5 +/− 0.4 billion platelets, had statistically significant positive outcomes at 6 months vs. control, whereas three studies with a low mean platelet dose of 2.3 +/− 0.4 showed no difference versus control (*p* < 0.01) [30]. These findings strongly suggest that a higher platelet dose may be correlated with better clinical results following PRP implantation for KOA. These data also strongly support the idea that concentration alone does not correspond to dosage, and it can be misleading [12,31]. The correct concentration calculation considers the baseline platelet concentration (which varies by patient), the volume of blood collected, and the final volume of the PRP product. As with any other therapeutic treatment, dosage should be standardized, referring to the absolute amount of platelets and any other components of PRP provided per injection, leading to the novel idea of a minimal effective platelet dose. Our data support the efficacy of a Neutrophil-Poor PRP containing a 4 billion platelet dose, comparable to the 5 billion dose used by Patel et al. [25], which observed a distinct pattern in the results between the two dose groups: a continuously improving trend in outcome scores from 3-month to 6-month follow-up in the 5.5 billion platelet dose group, while in the 2.5 billion platelet dose, the best scores were measured at 3 months with deterioration at 6 months, albeit still better than baseline. Our retrospective trial found the same trend in all three KL-graded groups. Interestingly, an optimal platelets dose superior to 3.5 billion has been supported for the first time by Everts et al. [13]. The effectiveness of different doses of PRP was studied in a variety of soft tissue studies, including tendinopathies, hamstring pathologies, meniscus lesions, and plantar fasciitis, relating, for the first time, the clinical outcome to the platelet dose [13,32] Studies with less than a 2 × 10^9^ dose of platelets injected showed a negative result, while studies where the dose exceeded 3.5 × 10^9^ platelets generally reported more positive results [13,32]. In keeping with the RESTORE randomized trial, patients with symptomatic KL2 and KL3 KOA treated with PRP intra-articular injections of 1.6 × 10^9^ platelets (325 × 10^3^/µL, 5 mL PRP), compared with saline placebo, showed no significant difference in symptoms or joint structure at 12 months [33]. Moreover, a recent study suggests a 10 billion platelet dose for a sustained therapeutic effect in KOA with a significant improvement in IKDC and WOMAC scores at one year follow-up [24]. For these reasons, we are evaluating switching from our actual LP-PRP protocol based on two PRP tubes drawing 10 mL of blood each, which ensures 4 billion platelets are injected, to two PRP tubes drawing 20 mL of blood with the same separator gel to harvest 8 billion platelet doses. An alternative strategy could be to repeat PRP treatment with 4 billion platelets after 12 months.

An explanation for the correlation between high-dose platelets or high-concentration folds (3–5X) and pain reduction, could be the high concentrations of serotonin released by platelet-dense granules [4]. PRP can reduce pain in patients treated for lateral epicondylitis and knee OA, as observed in a systematic review and meta-analysis [34].

In addition to the high platelet dose, regarding the biological profile, the PRP used in this study was neutrophil-depleted and red-cell-depleted, with a physiological concentration of mononuclear cells, and was non-activated (Table 2).

Regarding the presence of red blood cells, the data demonstrate that limiting or eliminating RBC content in PRP formulations is critical to avoid the detrimental effects of hemolysis and eryptosis on musculoskeletal tissues [18]. As a result, the PRP for these biologics should have been limited to no RBCs [35].

The influence of leukocyte concentration in PRP preparation is widely discussed [15,36]. Studies have demonstrated that leucocytes in PRP can harm cartilage, although Leukocyte-Poor PRP promotes chondrogenesis in vivo and superior functional outcomes [16,36,37,38]. Neutrophils may release inflammatory cytokines and matrix metalloproteinases (MMPs), which enhance proinflammatory and catabolic effects [39]. Positive outcomes after PRP treatment for OA are believed to be mostly related to the anti-inflammatory properties of PRP [9,10]. Furthermore, LR-PRP was found to increase the probability of local adverse effects compared to LP-PRP injection [40]. According to a recent meta-analysis, LP-PRP may be a more effective treatment for knee OA than LR-PRP [41]. These data are in accordance with the indications from meta-analysis, which suggested that the analgesic effect of LP-PRP was greater than that of LR-PRP [19]. However, not all leukocytes are the same: neutrophils are inflammatory cells that cause post-PRP treatment flares, whereas monocytes and macrophage phenotypes are regenerative cells that promote diverse mechanisms of action in tissue regeneration [4,42,43,44]. Based on extensive in vitro and in vivo data on peripheral blood mononuclear cells (PBMNCs), autologous preparations containing monocytes and lymphocytes are key components in tissue regeneration [45,46,47,48,49,50,51]. PBMNC showed enhanced cartilage repair, increased cell migration, and an immunomodulation effect both in animals and humans [52,53,54,55]. Interestingly, preliminary positive results in OA treatment by PBMNC autologous implants were observed [56,57,58,59,60]. Moreover, PBMNC, together with a high dose of platelets, may recruit other immune cells [51]. It has been well described that platelets in PRP should contribute to chemotactic cell migration and stimulate immunomodulatory activities [4], as observed for the first time by Sprugel, which indicates that PDGF, bFGF, and TGF beta induce granulation tissue through a secondary process mediated by macrophages and lymphocytes rather than a direct effect of the factors themselves [61]. To maximize this effect, GF release should be slow and last over time, especially considering the very short life of GFs (from minutes to hours) [62]. It has also been demonstrated that when pure PRP is given to tissue, it activates slowly upon encountering type I collagen in the absence of this preactivation [63]. Moreover, studies have shown that the proliferation of mesenchymal stem cells is increased five-fold by inactive, pH 7-buffered PRP [64]. More importantly, in a different study, it was shown that inactivated PRP enhanced the development of cartilage and bone both in vivo and in vitro, while activated PRP reduced chondrogenesis and osteogenesis [65]. Regarding safety, we did not observe any minor or major complications in our patient cohort. The major limitation of our study is the absence of a control group. Limitations of real-world data include the lack of randomization and the presence of potential biases and confounders. Analytic methods could help account for these limitations

Our real-world data, coming from routine clinical practice rather than protocol-driven trials, showed that inactivated, pH 7-buffered neutrophil- and RBC-depleted high-dose PRP had a positive outcome at 6 and 12 months in KL1-2-3-grade KOA patients, with no adverse effects. 

## 5. Conclusions

Our real-world data show that high-dose neutrophil- and RBC-depleted PRP, containing PBMNC at physiological levels, injected without prior activation is an effective treatment for KL1-KL3 KOA, showing an overall responder rate of 70%. It provides symptomatic relief, has the potential to reduce disease progression, and has sustained effects for up to 12 months. Moreover, this study supports evidence of a clinically effective dose PRP of 4 billion platelets, suggesting that the dose is the most critical parameter for sustained therapeutic effect. However, further studies are mandatory to evaluate if higher doses (8–10 billion) are more beneficial. Moreover, the complete absence of adverse events and side effects from using an autologous product uncontaminated by populations that can generate local inflammation should not be overlooked. This treatment can, therefore, be recommended for patients with KOA of varying degrees and can also be repeated over time. Furthermore, it remains a viable alternative for patients who do not respond to other conservative treatments or for patients who cannot or do not wish to undergo surgery.

## Figures and Tables

**Table 1:** Patient characteristics.

	*n* = 212
Age (mean +/− SD) Range (years)	56.09 +/− 9.23 19–89
Gender Male n (%) Female n (%)	42% 58%
BMI (kg/m^2^) Range BMI	25.3 +/− 4.1 23–28.3
Side	
Left/Right Knee (%)	44%/56%
KL Grade	
Grade 1 n (%)	21 out 212 (9.9%)
Grade 2 n (%)	127 out 212 (59.9%)
Grade 3 n (%)	64 out 212 (30.2%)
Basal Platelets (×10^3^/µL)	225 +/− 37
VAS score	6.49 +/− 1.15
WOMAC score	41.98 +/− 6.8

KL, Kellgren–Lawrence

**Table 2:** Biological profile of injected PRP (n = 60).

	Blood	PRP
Blood volume/PRP volume *	20 mL	4.1 +/− 0.3
Platelet conc. (10^3^/µL)	235 +/− 37	960 +/− 108
Platelet dose (billions 10^9^)	4.7 +/− 0.79	3.9 +/− 0.2
WBC (10^3^/µL)	10.3 +/− 2.3	3 +/− 0.22
Granulocyte (10^3^/µL)	7.5 +/− 0.89	0.5 +/− 0.05
Lymphocytes (10^3^/µL)	2.7 +/− 1.6	2.1 +/− 0.8
Monocytes (10^3^/µL)	0.78 +/− 1.8	0.42 +/− 0.49
PB-MNC (10^3^/µL)	3.56 +/− 1.2	2.38 +/− 0.98
RBC (10^6^/µL)	5.40 +/− 2.9	0.053 +/− 0.01
Platelet conc. fold		4×
Platelet recovery rate %		82.1 +/− 0.4
% Mononuclear cells/WBC		80%

* n = 60 (n = 20 for each KL group). Two TropoCells PRP tubes for each patient were used for a total of 20 mL of blood withdrawal and 4 mL of injected PRP.

**Table 3:** Responder rate for OMERACT-OARSI responder criteria at 3, 6, and 12 months.

	Non-Responder	Responder	Patients	Responder Rate
Overall				
3 months	61	151	n = 212	71.2%
6 months	56	156	n = 212	73.6%
12 months	66	147	n = 212	69.3%
KL Grade 1				
3 months	6	15	n = 21	71.4%
6 months	5	16	n = 21	76.2%
12 months	6	15	n = 21	71.4%
KL Grade 2				
3 months	36	91	n = 127	71.7%
6 months	33	94	n = 127	74.0%
12 months	39	88	n = 127	69.3%
KL Grade 3				
3 months	19	45	n = 64	70.3%
6 months	18	46	n = 64	71.9%
12 months	20	44	n = 64	68.8%

Two TropoCells PRP 11 mL tubes for each patient; 4 mL PRP injected.

**Table 4:** Clinical outcome: VAS and WOMAC at 3, 6, and 12 months.

	Basal—Pretreatment	3 m	6 m	12 m
VAS pain				
Overall	6.49 +/− 1.15	4.3 +/− 1.8 *	2.69 +/− 0.98 *	3.79 +/− 0.78 *
KL1	6.02 +/− 1.09	4.66 +/− 1.23 *	2.81 +/− 0.6 *	3.81 +/− 0.3 *
KL2	6.41 +/− 0.65	4.15 +/− 0.98 *	2.6 +/− 0.53 *	3.65 +/− 0.89 *
KL3	6.58 +/− 1.09	4.99 +/− 1.2 *	2.4 +/− 0.99 *	3.48 +/− 1.1 *
WOMAC				
Overall	41.98 +/− 6.8	36.55 +/− 13.5 *	34.9 +/− 11.8 *	39.3 +/− 8.05
KL1	40.65 +/− 11.9	36.99 +/− 9.5 *	35.8 +/− 12.3 *	38.2 +/− 9.99
KL2	41.65 +/− 9.5	35.05 +/− 9.99 *	34.1 +/− 14.0 *	38.1 +/− 8.67
KL3	40.65 +/− 3.9	36.18 +/− 4.89 *	35.8 +/− 9.06 *	40.13 +/− 12.04

Data are reported as mean ± SD. VAS, Visual Analog Scale; WOMAC, Western Ontario and McMaster Universities Osteoarthritis Index. * Significant differences between groups for that time point (*p* < 0.001; Wilcoxon signed-rank test).

## Data Availability

For this study, the electronic clinical records of this cohort’s patients were reviewed retrospectively. The data are not publicly available due to the protection of patients’ privacy.
